# The Effects of Patients’ Health Information Behaviors on Shared Decision-Making: Evaluating the Role of Patients’ Trust in Physicians

**DOI:** 10.3390/healthcare13111238

**Published:** 2025-05-24

**Authors:** Mingming Song, Joel Elson, Christian Haas, Sharon N. Obasi, Xinyu Sun, Dhundy Bastola

**Affiliations:** 1 School of Interdisciplinary Informatics, University of Nebraska Omaha, Omaha, NE 68182, USA; jselson@unomaha.edu (J.E.); christianhaas@unomaha.edu (C.H.); 2 Department of Counseling, School Psychology and Family Science, University of Nebraska at Kearney, Kearney, NE 68849, USA; obasis2@unk.edu; 3 Department of Biostatistics, University of Nebraska Medical Center, Omaha, NE 68198, USA; xsun@unmc.edu

**Keywords:** shared decision-making, trust, health information retrieval, social media, emotional response, patient-centered care, patient engagement

## Abstract

**Background/Objectives**: The availability of accessible health information outside of traditional healthcare settings has transformed patient engagement in shared decision-making (SDM) with healthcare providers. However, the challenge of navigating misinformation complicates SDM, highlighting the critical role of trust, especially when patient-achieved information conflicts with professional advice. This study examines the association between patients’ health information behavior (HIB) and SDM, emphasizing the role of patients’ trust in healthcare providers. **Methods**: Utilizing data from the Health Information National Trends Survey (HINTS), this research explores how trust mediates the relationship between HIB and SDM. We conducted factor analysis, mediation analysis, and moderated mediation analysis to assess our hypotheses. **Results**: Factor analysis identified two main HIB dimensions: emotional responses and utilization of social media. Emotional responses positively influenced SDM, enhancing trust and decision-making involvement. In contrast, utilization of social media negatively influenced SDM through decreased trust. Mediation analysis confirmed trust in physicians as a crucial mediator, particularly when emotional responses foster trust and engagement. Moderated mediation showed that high healthcare quality amplified the positive mediation effects of trust, underscoring its role in effective SDM. **Conclusions**: This study highlights the significant role of trust in enhancing patient engagement in SDM through HIB. High perceived healthcare quality also strengthens trust, improving SDM outcomes. The study contributes to the literature by providing a comprehensive analysis of the interplay between HIB, trust, and SDM, suggesting that enhancing patient-centered care requires fostering trusted patient–physician relationships.

## 1. Introduction

The availability of easily accessible health information outside of traditional healthcare sources is changing how patients engage in shared decision-making (SDM) with healthcare providers. SDM is a collaborative decision-making process between a healthcare provider and patient [1,2], with healthcare professionals guiding relatively uninformed patients [3,4]. The patient, working with their healthcare provider, would come to a shared decision around a personally appropriate healthcare plan. Here, the trust in the healthcare provider is critical as their guidance helps patients to sort through information and fosters communication in the SDM, empowering patients.

In today’s digital age, patients are taking more initiative in their health information behavior (HIB), actively seeking, processing, and managing health information [5,6]. While the abundance of health information is leading to new HIB and empowers patients with increased knowledge, it simultaneously brings new challenges in SDM. Patients and physicians must now navigate through an increasing volume of misinformation and irrelevant health information [7,8]. In this context, patients’ trust in physicians becomes crucial in shaping how HIB relates to SDM. Prior research suggests that trust influences whether patients act on the information they achieve and how openly they collaborate with providers during decision-making, especially when conflicts occur between self-gathered information and that provided by physicians [9,10]. Rather than simply replacing certain information, trust may help patients feel more comfortable discussing uncertainties and reconciling different sources of information during SDM. This study explores how trust contributes to this process of patients’ health information behavior.

Although trust’s role in SDM is acknowledged, understanding its transition from HIB remains limited. Previous research explored trust’s impact in specific contexts but lacks broader insights on how patient HIBs associate with SDM [11,12]. There is a need to investigate how the role of patients’ trust in physicians influences the transition from HIB to SDM, including assessing the relationship and underlying mechanisms. Specifically, this study addresses two overarching research questions: (1) How do different health information behaviors affect shared decision-making? and (2) What role does trust play in linking patients’ health information behavior to shared decision-making?

## 2. Theoretical Background and Hypothesis Development

### 2.1. Shared Decision-Making (SDM) and Health Information Behavior (HIB)

SDM ensures patient-centered, evidence-based decisions tailored to individual needs, empowering patients in their healthcare [13,14]. Additionally, SDM fosters a trusted relationship between patients and physicians, essential for high-quality healthcare delivery [15]. However, the effectiveness of SDM can encounter barriers, such as patients’ lack of expert domain knowledge and difficulty in identifying useful health information [16]. Barriers may prevent patients from fully understanding their treatment options, recognizing how these options align with their personal needs, and assessing potential associations with their quality of life. Consequently, patients may find it challenging to contribute effectively to the decision-making process.

Effective SDM may benefit from patients’ active engagement in HIB, which encompasses finding, understanding, and managing health-related information, an evolution from Health-Information-Seeking Behavior (HISB) [13]. In today’s digital age, HIB has evolved with the rise of health information systems and diverse information sources, as patients navigate online resources [14]. Studies show high internet usage for health purposes, indicating the importance of being well-informed for effective SDM [17,18]. Effective HIB enables patients to actively contribute to healthcare discussions and decision-making, ultimately improving healthcare outcomes [19]. This is because well-informed patients are better prepared to have meaningful discussions with their doctors, improving the SDM process with more effective engagement. For example, patients actively learning about health topics are more inclined to discuss treatment options with their healthcare providers [20]. Building on the understanding that effective HIB could play a crucial role in facilitating SDM, we formulated our first hypothesis (H1) to investigate this association:

**H1.**  *Patients’ health information behavior (HIB) is significantly associated with shared decision-making (SDM).*

### 2.2. The Role of Patients’ Trust in Physicians

Trust in the patient–doctor relationship is recognized as essential for healthcare [21,22]. From the perspective of patients interacting with physicians, trust is defined and utilized in this study involving two main aspects:

1. Patients’ Trust in Physicians: patients’ confidence in their physicians and the information they provide, believing it to be accurate, reliable, and in their best interest [23,24].
2. Patients’ Trust in Information Sharing: patients’ health data sharing with their physicians, trusting them to use them appropriately and maintain confidentiality [25,26].

Patients’ trust in physicians is critical for effective health communication and SDM, especially in navigating the complexities of conflicting or misleading information. Trust encourages open conversation, enabling patients to discuss their findings and concerns with their physicians, further enhancing the quality of healthcare outcomes [26]. Furthermore, a strong foundation of trust empowers patients to actively engage in SDM [27,28]. Patients who have a high level of trust in their physicians are more likely to engage in the shared decision-making process, underscoring a direct link between trust and the willingness to engage in SDM. Therefore, patients’ trust in physicians may play a crucial role in bridging patients’ independent health information activities (such as HIB) with collaborative healthcare processes (such as SDM).

**Figure 1:**
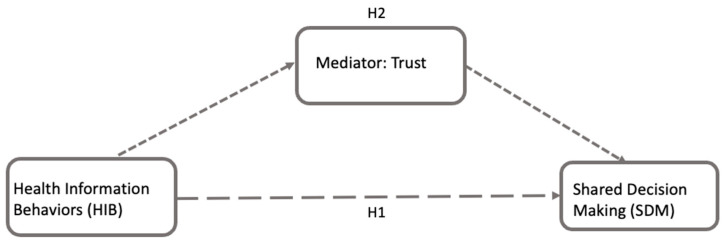
This figure shows the conceptual framework for H1 and H2, exploring the relationships between HIB, trust, and SDM using regression-based mediation analysis. H1 suggests a direct path from HIB to SDM. That is, the way patients seek, process, and manage health information may be directly associated with their involvement in the SDM. H2 represents an indirect (mediated) path, where trust mediates the influence of HIB on SDM. All arrows are dashed to indicate theoretical pathways rather than observed statistical paths.

While the importance of trust is obvious, its specific roles in the relationship between HIB and SDM need further exploration. Expanding the methodologies used in previous studies [5], which explored the links between health-related social media use, patient-centered communication, and health outcomes, this study investigates how trust influences the dynamic interplay between HIB and SDM with mediation analysis (Figure 1). Specifically, it studies whether trust mediates the relationship between HIB and SDM (H2), perhaps acting as an intermediary mechanism that could explain how HIB leads to SDM. Determining the mediating effects of trust will be useful for understanding, and ultimately improving, SDM for health-related decisions. Hypothesis H2 is formulated as follows:

**H2.**  *The role of patients’ trust in physicians, including trust in information from physicians (H2.1) and sharing health data with physicians (H2.2), mediates the relationship between patients’ health information behavior (HIB) and shared decision-making (SDM).*

### 2.3. Robustness Checking

**Figure 2:**
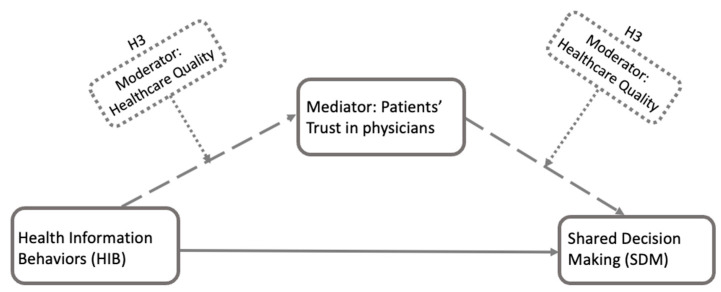
This is the conceptual framework for H3 based on H1 and H2. As a robustness-checking process, H3 explores whether the patients’ perceived healthcare quality moderates the strength or direction of the potential mediation relationship from HIB to SDM through mediator trust using regression-based moderated mediation analysis. Dashed arrows represent hypothesized conceptual paths. Dashed-line boxes indicate moderator variables, which influence the links between HIB and trust, and between trust and SDM.

To robustly examine the influence of trust, this study assesses whether trust independently influences the relationship between HIB and SDM. Prior research indicates that patients’ trust in their physicians is positively associated with their overall satisfaction and perception of healthcare quality [29,30]. This association suggests that, even if trust plays a mediating role, it may not independently influence the path from HIB to SDM. The strength of this potential mediation may be influenced by other factors, such as perceived healthcare quality. Therefore, in our moderated mediation model (Figure 2), if trust serves as a mediator (H2) in the transition from HIB to SDM, perceived healthcare quality is proposed to moderate (H3) this mediation, influencing the effect of trust in this pathway.

**H3.**  *If trust mediates the relationship between health information behavior (HIB) and shared decision-making (SDM), then perceived healthcare quality acts as a moderator in this mediation.*

## 3. Materials and Methods

### 3.1. Data Descriptions

This study utilized data from the Health Information National Trends Survey 6 (HINTS 6), a national cross-sectional survey designed to assess the American public’s health information behavior [24,25]. As the most recent cycle, HINTS 6 provides up-to-date insights into how participants access, use, and perceive health information. Data collection for the HINTS 6 cycle began in March 2022 and concluded in November 2022. The survey captured responses from 6252 participants.

### 3.2. Measurements

#### 3.2.1. Independent Variable (IV): Health Information Behavior (HIB)

For this study, HIB was initially measured using 18 items from the HINTS 6 dataset. These items capture a broad range of patient behaviors and reactions to health information, such as source usage, emotional responses, frequency, and reliance on digital health tools. A detailed description of these items (I1–I18), a factor analysis (detailed later in the analysis section), and the corresponding response scales are provided (Appendix A), allowing for an in-depth analysis of how individuals interact with health information. Because the HINTS dataset was not originally designed to measure health information behavior as a single construct, we employed exploratory factor analysis (EFA) to empirically identify coherent dimensions from the 18 selected items. This approach aligns with established practices in health research, where EFA is employed to identify latent constructs within survey data. For instance, previous studies have utilized EFA to validate health behavior scales, confirming its applicability in similar contexts [31]. Our selection of these 18 items was guided by their relevance to HIB and their inclusion in prior research, ensuring a comprehensive assessment of the construct.

#### 3.2.2. Dependent Variable (DV): Shared Decision-Making (SDM)

The dependent variable in this study, SDM, was indicated by respondents’ self-reported involvement in healthcare decisions. Specifically, participants were asked, ‘*In the past 12 months, how often did your doctors, nurses, or other health professionals involve you in decisions about your health care as much as you wanted?*’ The frequency of their involvement was measured using a four-point Likert scale with options ranging from ‘*Always*’ to ‘*Never*’.

#### 3.2.3. Patients’ Trust in Physicians

Patients’ trust in physicians was captured through two distinct concepts, as we previously described during hypotheses’ development. These concepts are represented by specific questions that the authors carefully selected from the HINTS 6 survey for analysis.

- Concept 1: Trust in Information from Physicians (T1). Representing patients’ trust in the information provided by physicians, one survey question was selected from the survey: “*In general, how much would you trust information about cancer from a doctor?*” This was measured using a four-point Likert scale with options ranging from ‘*A lot*’ to ‘*Not at all*’. This question was intended to assess the level of confidence that patients have in their physician as a source of health information, focusing on the general trust patients have in their physicians.
- Concept 2: Trust in Information Sharing with Physicians (T2). Representing patients’ trust in sharing digital health-related data with their physicians, two questions were selected to represent this dimension of trust: (1) “*Would you be willing to share health data from your wearable device with your health care provider?*” and (2) “*Have you shared health information from either an electronic monitoring device or smartphone with a health professional within the last 12 months?*” These questions were measured using a binary scale, yes or no. The questions aimed to quantify the level to which patients are comfortable with and trust their physicians in handling and maintaining the confidentiality of their personal health information.

### 3.3. Data Analysis

Data analysis was conducted using R, starting with exploratory factor analysis (EFA) to categorize HIB variables into coherent groups. Linear regression analysis was then utilized to test Hypothesis 1, which explored the association between HIB and SDM. For Hypothesis 2, mediation analysis was conducted to investigate how patients’ trust in physicians affects these relationships. Finally, moderated mediation analysis was implemented to assess the potential moderating effects on the mediation relationship.

Prior to data analysis, multiple imputation techniques were evaluated to prepare the dataset and address any issues related to missing or inapplicable data. k-Nearest Neighbors (KNN) Imputation was ultimately selected based on its lower Residual Standard Error (RSE) (0.05) and higher Adjusted R-Squared (2.39) when compared to Random Forest Imputation (RSE = 0.06, Adjusted R-Squared = 2.22) and Multiple Imputation with MICE (Multivariate Imputation by Chained Equations) (RSE = 0.06, Adjusted R-Squared = 2.33) [32]. This suggested that KNN provided a better fit and greater ability to explain data compared with other imputation methods (Appendix B). With the data thus prepared, the study proceeded to test the hypotheses, focusing on the dynamic interplay between HIB, trust, healthcare quality, and SDM.

#### Exploratory Factor Analysis (EFA)

**Figure 3:**
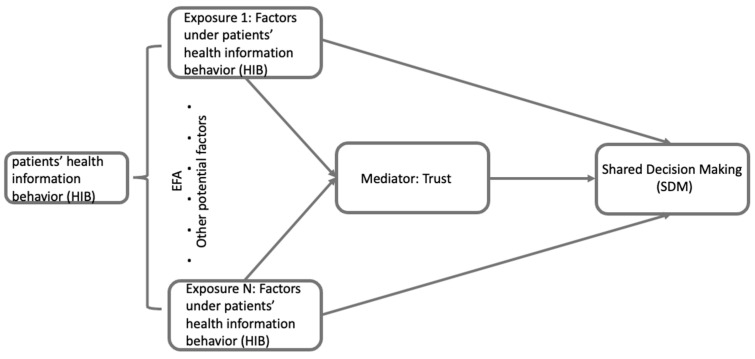
This is the conceptual framework for the exploratory factor analysis (EFA). The figure shows the process of identifying different categories within HIB through EFA and how these categories will be applied for examining the hypotheses of this study.

Given the multifaceted nature of HIB and the diverse items included in the HINTS 6 dataset to measure it, EFA was utilized to categorize the 18 survey questions related to HIB into coherent, interpretable groups that reflected distinct dimensions of HIB (Figure 3). The resulting factor groups were then used as separate variables in the mediation analysis. The first factor, emotional response, was reverse-coded so that higher scores reflected more positive emotional experiences during health information behavior (e.g., less frustration, greater satisfaction). This coding direction was applied to ensure consistency in interpretation across all variables. The second factor, utilization of social media, captured the extent of patients’ engagement with social media platforms for health-related information. These factors are treated as distinct dimensions of HIB in the subsequent analyses to allow clearer interpretation of their theoretical and practical relevance.

## 4. Results

### 4.1. Demographics

Of the 6252 participants in this study, more than 60% were female (62.4%, n = 3899) and more than 50% were non-Hispanic White (51.2%, n = 3203). Most of the participants had some college and higher levels of education (73.5%, n = 4594) and did not have cancer (82.7%, n = 5168). Demographic variables were controlled in all the analyses of this study (Appendix C).

### 4.2. Factor Analysis

The iterative process of factor analysis resulted in two factor groups, each including four items from the HIB measures. Items with low loadings and weak impact on the variables were excluded, resulting in a refined set of factors. Analysis of the item content within these groups revealed two clear categories (Table 1), labelled as (1) emotional responses during HIB (eigenvalue = 2.88; factor loading = 0.7–0.9), and (2) utilization of social media during HIB (eigenvalue = 2.07; factor loading = 0.5–0.7). These items were reverse-coded where necessary, so that higher scores consistently reflected more positive emotional responses or more utilization of social media during information seeking. A careful review of the items in each category confirmed their conceptual coherence to justify their collective use in further analyses (Appendix D).

### 4.3. Linear Regression Analysis

**Figure 4:**
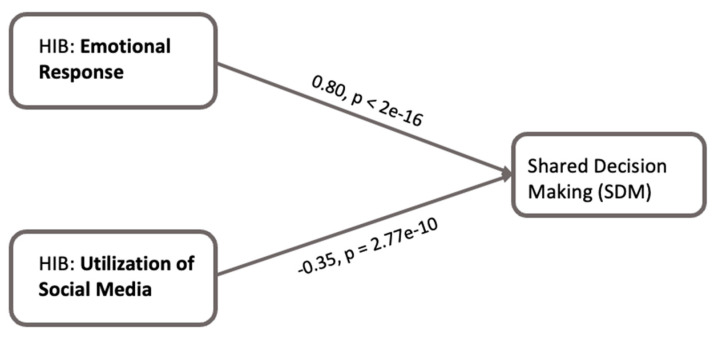
Conceptual framework for effect (total effect) of HIB (including emotional response and utilization of social media) on SDM. The figure illustrates the findings for H1 based on two categories from the factor analysis results. The emotional response of patients during HIB indicates a statistically significant positive association with the involvement of SDM. The utilization of social media for health-related purposes suggests a significant negative association with the involvement of SDM.

Linear regression analysis was utilized to evaluate the effect of HIB (including emotional response and utilization of social media) on the outcome variable (Figure 4) while controlling for various demographic factors and cancer status. The results suggest a significant positive association between emotional response during HIB and SDM, as indicated by a positive coefficient (β = 0.803 *p* < 2 × 10^−16^). Conversely, the utilization of social media within HIB showed a significant negative relationship with SDM, marked by a negative coefficient (β = −0.348, *p* = 2.77 × 10^−10^). Therefore, we support our hypothesis that HIB is significantly associated with SDM (H1). This means that, when patients feel less overwhelmed and more emotionally satisfied during their information search, they are more inclined to work with their providers in making decisions. However, if they rely too much on social media, they may be exposed to misinformation or develop conflicting views, which could lead to reduced involvement in decision-making.

### 4.4. Mediation Analysis

To test Hypothesis 2, we employed mediation analysis to explore how patients’ trust in physicians mediates the relationship between different categories of HIB and SDM. Specifically, we examined the separate mediation effects of trust in physician-provided information (T1) and trust in sharing information with physicians (T2) on the relationships between SDM and (a) emotional responses during HIB and (b) social media utilization during HIB.

### 4.5. Patients’ Trust in Information Provided by Physicians (T1)

**Figure 5:**
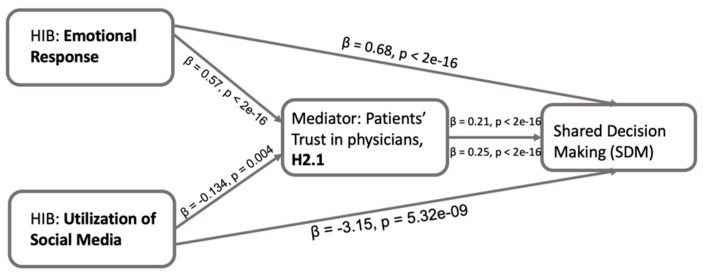
Conceptual framework for the direct and indirect effects of HIB on SDM via patients’ trust in physicians (H2.1). The figure shows regression-based mediation analyses where patients’ trust in physicians or information from physicians has a mediating role in the path from patients’ HIB (including emotional response and utilization of social media) to SDM.

Considering the patients’ trust in physicians as a potential mediator, the evidence of the direct and indirect effects of HIB on SDM, as assessed by their emotional response, was estimated by conducting linear regression models (Figure 5). In the mediator model, the emotional response showed a significant positive effect on patients’ trust in physicians (β = 0.573, *p* < 2 × 10^−16^). In the outcome model, both emotional response (β = 0.683, *p* < 2 × 10^−16^) and patients’ trust in physicians (β = 0.209, *p* < 2 × 10^−16^) showed significant positive effects on SDM. Additionally, a nonparametric bootstrapped mediation analysis revealed a partial mediation effect of patients’ trust in physicians (Table 2). Furthermore, the Average Causal Mediation Effect (ACME) for the indirect effect (β = 0.120, *p* < 2 × 10^−16^) suggested that patients’ trust in physicians partially mediates (positively) the relationship between emotional response and SDM. The proportion mediated was approximately 14.93%. In simpler terms, when patients have a more positive emotional experience while seeking and processing information, it enhances their trust in their physicians, which makes them more likely to actively engage in healthcare decisions.

Similarly, the social media utilization in the linear regression model showed a significant negative effect on patients’ trust in physicians (β = −0.134, *p* = 0.004). In the outcome model, utilization of social media showed a significant negative effect on SDM (β = −3.151, *p* = 5.322 × 10^−9^), while patients’ trust in physicians showed a significant positive effect on SDM (β = 0.248, *p* < 2 × 10^−16^). Additionally, a nonparametric bootstrapped mediation analysis revealed a partial mediation effect of patients’ trust in physicians (Table 2). The ACME for the indirect effect (β = −0.033, *p* = 0.008) suggested that patients’ trust in physicians partially mediates (negatively) the relationship between emotional response and SDM. The proportion mediated was approximately 9.53%.

### 4.6. Patients’ Trust in Sharing Information with Physicians (T2)

**Figure 6:**
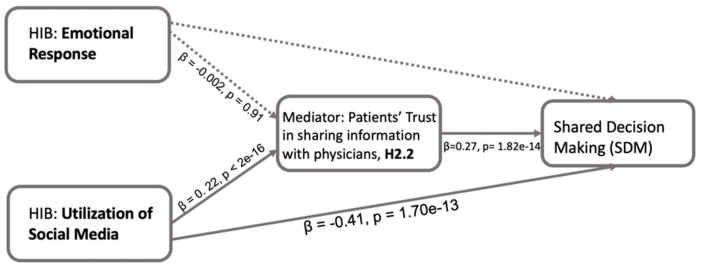
Mediation model examining the direct and indirect effects of HIB on SDM through patients’ trust in sharing information with physicians (H2.2). This figure presents regression-based mediation analysis results. Solid arrows represent statistically significant paths. Dashed arrows indicate non-significant paths based on the regression analysis. Path coefficients and *p*-values are displayed along each arrow. The model suggests that patients’ trust in sharing information with physicians mediates the relationship between their utilization of social media and SDM, while no significant mediation is found between emotional response and SDM via trust.

The mediation model exploring the potential mediating role of trust in sharing information with physicians (T2) in the relationship between emotional response and SDM did not indicate significant results (Figure 6). The regression analysis did not show a significant effect of emotional response on T2 (β = −0.002, *p* = 0.910). Given the lack of a significant association, the mediation analysis was not pursued further for this pathway.

In the linear regression model, the utilization of social media showed a significant positive effect on trust in sharing information with physicians (β = 0.223, *p* < 2 × 10^−16^). In the outcome model, utilization of social media showed a significant negative effect on SDM (β = −0.409, *p* = 1.70 × 10^−13^), while patients’ trust in sharing information with physicians showed a significant positive effect on SDM (β = 0.274, *p* = 1.82 × 10^−14^). Additionally, a nonparametric bootstrapped mediation analysis revealed a partial mediation effect of patients’ trust in information sharing with physicians (Table 2). The ACME for the indirect effect (β = 0.061, *p* < 2 × 10^−16^) suggested that patients’ trust in physicians partially mediates (positively) the relationship between emotional response and SDM. The proportion mediated was −17.52%.

The results for Hypothesis 2 indicate that patients’ trust in physicians partially mediates the relationship between both emotional response and utilization of social media during HIB and SDM, confirming H2.1. On the other hand, for H2.2, while the health data sharing with physicians did not mediate the effect of (a) emotional response on SDM, it did show a mediating role for (b) the utilization of social media, indicating a selective mediation effect within the HIB dimensions.

### 4.7. Moderated Mediation Analysis

In the examination of Hypothesis 3, moderated mediation models were constructed to explore the role of perceived healthcare quality in the mediation relationship between HIB and SDM with trust acting as a mediator. The lavaan package was used in R to estimate the model.

**Figure 7:**
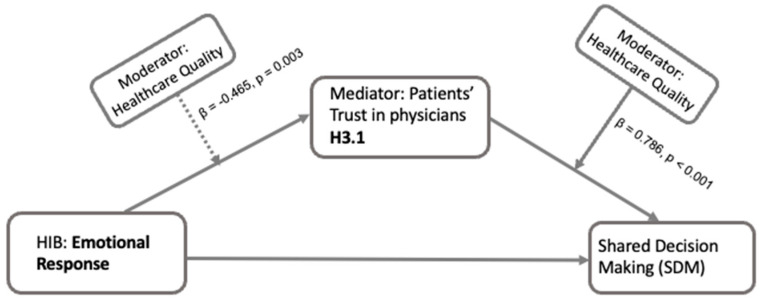
Moderated mediation effect of healthcare quality on the relationship between emotional response and SDM through patients’ trust in physicians (H3.1). The figure shows that the mediation effect of patients’ trust in physicians within the path from patients’ emotional repose during HIB to their involvement in SDM is moderated by patients’ perceived quality of healthcare.

For the emotional response aspect of HIB and patients’ trust in physicians (T1), the results revealed a significant interaction between emotional response and perceived healthcare quality in predicting trust in physicians (β = −0.465, *p* = 0.003), suggesting that the effect of emotional response during HIB on trust varies depending on the level of perceived healthcare quality (Figure 7). Furthermore, perceived healthcare quality positively influenced trust in physicians (β = 0.786, *p* < 0.001), suggesting that higher perceived quality correlates with increased trust. The moderated mediation effect was also significant (β = −0.079, *p* = 0.004, Table 2), showing that the mediation effect of trust is moderated by the perceived quality of healthcare.

**Figure 8:**
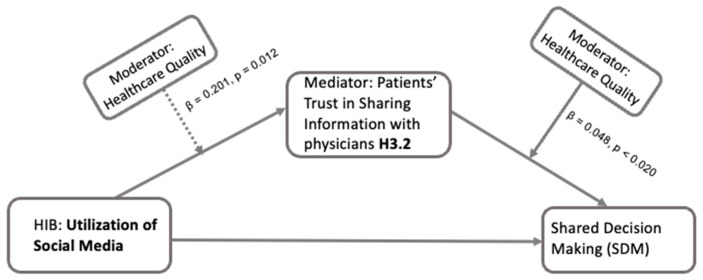
Conceptual framework for moderated mediation effect of healthcare quality on the relationship between utilization of social media and shared decision-making through patients’ trust in sharing information with physicians (H3.2). The figure shows regression-based moderated mediation analyses where the mediation effect of patients’ trust in sharing information with physicians within the path from patients’ utilization of social media during HIB to their involvement in SDM is moderated by patients’ perceived quality of healthcare.

For the utilization of social media and its effect on patients’ trust in sharing information with physicians (T2), the results showed (Figure 8) that the interaction with perceived healthcare quality was significant (β = 0.201, *p* = 0.012), suggesting an enhancing effect of perceived healthcare quality on trust influenced by social media use. Furthermore, perceived healthcare quality positively influenced this type of trust (β = 0.048, *p* < 0.020), suggesting that higher perceived quality correlates with increased trust. Finally, the moderated mediation effect was also significant (β = 0.087, *p* = 0.013), showing that the mediation effect of trust is moderated by the perceived quality of healthcare.

Table 2 provides an overview of the combined results, including mediation analysis results for Hypothesis 2 and Hypothesis 3. The results demonstrate that perceived healthcare quality has a selective significant moderating role in the relationships between different dimensions of HIB and SDM, mediated by trust.

The essential role of trust is clearly identified by these analyses. Based on the associations observed between multiple dimensions of HIB and SDM, various aspects of patients’ trust in physicians have been fully explored through mediation analyses. Specifically, our findings indicate that patients’ trust in physicians or the information that comes from physicians partially mediates the associations between both emotional response and social media utilization during HIB and SDM. Additionally, patients’ trust in sharing health-related data with physicians partially mediates the association between social media utilization during HIB and SDM. Furthermore, with the moderator role of patients’ perception of the healthcare quality they received, the mediation effects of trust are moderated, shaping the overall effectiveness of SDM. These findings highlight the complex interplay between HIB, trust, and healthcare quality in optimizing patient involvement in healthcare decisions.

## 5. Discussion

This study enhances our understanding of trust in patient–physician relationships and how it functions within the pathway from patients’ HIB to SDM. Consistent with previous research [33,34], our results emphasize the significant association between HIB and patient engagement in SDM, supporting Hypothesis 1 (H1). This aligns with theoretical frameworks of patient empowerment that highlight emotional readiness as foundational for effective SDM [35]. Moreover, the negative association between social media utilization and SDM aligns with prior studies about the potential online misinformation from social media that undermines trust and SDM [7,36]. Additionally, the finding for our second hypothesis (H2) (i.e., the role of patients’ trust in physicians) reveals the partial mediation role of patients’ trust in physicians between HIB and SDM. This supports recent relational models of SDM that emphasize the central role of interpersonal trust in enabling informed and shared decisions [37,38]. Finally, the findings for Hypothesis 3 (H3) highlight the importance of perceived healthcare quality as a moderating factor in the relationships established by H2. Specifically, the moderated mediation results elaborate that high perceived healthcare quality strengthens the positive association between emotional responses and trust, which, in turn, enhances SDM. This underscores the layered complexity of trust, quality perceptions, and information behavior in shaping patient participation in healthcare decisions [39].

These findings from our mediation and moderated mediation analysis offer both theoretical and practical implications for improving patient–physician communication and emphasizing the importance of trusted relationships. The mediating role of trust suggests that trust is not just a result of positive healthcare interactions but also a key mechanism that connects patients’ emotional experiences and information-seeking and processing experiences to their engagement in SDM. Trust enables patients to take a more active role in decisions that directly influence their care. Additionally, the result that perceived healthcare quality strengthens the emotional trust pathway highlights the value of maintaining high-quality care—not only to improve clinical outcomes, but also to support patients’ confidence during SDM. These findings point to future research opportunities for designing trust-building interventions that address patients’ emotional responses and perceptions of care. In practice, physicians may benefit from communication strategies that are aligned with patients’ emotional needs and preferences, which facilitate patients’ readiness to participate in SDM. The complex mediation effects of trust across different aspects of HIB, including emotional responses and social media utilization (H2), are discussed. We elaborate on how patients’ perceived healthcare quality influences these mediation effects (H3). Additionally, to allow for an enhanced understanding of how each factor contributes to the overall dynamics between patients and physicians, the discussion is organized below to differentiate the effects by the specific aspects of HIB.

### 5.1. Mediating Effects of Trust

While this study began with a broad conceptualization of HIB, our exploratory factor analysis (EFA) identified two distinct dimensions during HIB: emotional response and social media utilization. These two factors reflect different types of health information engagement and were analyzed and interpreted separately in our mediation and moderated mediation models. This differentiation allowed for a more nuanced understanding of how each dimension uniquely relates to trust and SDM.

Figure 4 shows that positive emotions during HIB are strongly linked to increased patient involvement in SDM, suggesting that fostering positive feelings like satisfaction and empowerment in HIB can encourage patients to take a more active role in healthcare decision-making. This emphasizes the importance of emotions in patient engagement and patient-centered care [35]. Moreover, patients’ trust in physicians partially mediates the link between emotional responses during HIB and SDM (Figure 5). This further emphasizes that patients experiencing positive emotions during HIB are more likely to trust their physicians or the information provided by their physicians, leading to greater participation in SDM. However, health data sharing with physicians did not show a mediating effect (Figure 6), possibly due factors such as privacy concerns and the relevance of information [40,41]. Patients may hesitate to share certain details due to fear of judgment or stigma, regardless of their trust in the physician. This suggests that patients’ trust in the information provided by physicians plays a more significant role in mediating the association between emotional response during HIB and SDM than patients’ trust in sharing personal health information. Further, the patients’ positive emotional responses enhance their trust in physicians, which, in turn, facilitates patients’ active engagement in SDM.

Our study revealed a notable negative association between social media use during HIB and the frequency of shared decision-making (SDM) (Figure 4). This observation suggests that increased reliance on social media for health information might reduce the frequency of patient involvement in decision-making. Despite prior research indicating a positive association between online healthcare information and SDM [42], social media use may differ due to concerns about reliability and misinformation [43]. Furthermore, trust (both in physicians and in sharing information with them) partially mediates the relationship between social media use during HIB and SDM (Figure 5 and Figure 6). Increased social media use is linked to reduced trust in physicians and decreased SDM. One explanation is that patients may encounter overwhelming and misleading health information on social media, complicating trust-building with physicians [44]. Additionally, social media might foster false confidence in self-diagnosis, reducing reliance on physicians [45]. While online health information empowers patients, guidance is needed to navigate and interpret it effectively.

Regarding trust in sharing health data with physicians and its relationship with SDM, the utilization of social media appears to increase trust in sharing information but does not boost active participation in SDM. A −17.52% mediated proportion suggests a complex, potentially inverse relationship between the utilization of social media during HIB and SDM through trust (Table 2). Social media may inform patients, making them more willing to discuss health issues with physicians [46]. However, misinformation from social media could reinforce preconceived beliefs, hindering SDM. Trust in sharing information can mitigate the negative association between social media use and SDM, emphasizing the importance of effective health communication and trusted patient–physician relationships in facilitating SDM. In addition, while we interpret sharing information as an indicator of trust, it is also possible that this behavior reflects personal preferences during clinical interactions. This alternative interpretation highlights the complexity of behavioral indicators used to measure trust. Future research should explore the varied motivations behind data-sharing behaviors to further explore what drives patients to share personal health information.

Although this study primarily positions trust as a mediator, it is also possible that patients’ baseline trust levels influence their choice of information sources. For instance, individuals with lower trust in physicians or limited access to timely medical advice might be more inclined to rely on online or social media. While our cross-sectional design limits causal interpretation, future longitudinal studies could help clarify whether trust also functions as an antecedent to health information behavior.

### 5.2. Trust and Patients’ Perceived Healthcare Quality

Patients’ trust in physicians can be influenced and changed by various factors, such as information exposure [47] and interactions with healthcare providers [48], before the final healthcare outcome is achieved. In addition to discussing the above findings based on the mediation models, we offer another significant result relating to Hypothesis 3 (H3) that explores the moderator role of patients’ perceived healthcare quality in the relationship between HIB and SDM, with trust as the mediator.

For emotional responses in HIB, there is an interaction with perceived healthcare quality when the mediator is patients’ trust in physicians (Figure 7). Specifically, when healthcare quality is perceived as high, emotional responses seem to have less of an association with trust in physicians. This suggests that if patients perceive healthcare quality to be high, their emotions during HIB play a lesser role in building trust in their physicians. This finding aligns with the idea that patients’ overall satisfaction and expectations in healthcare can influence confidence and building trust in healthcare providers [39]. Moreover, patients’ perceived healthcare quality has a direct positive influence on trust in physicians. This indicates that higher perceived healthcare quality is directly associated with greater trust in physicians [33]. Our finding underscores that, at least for emotional response during HIB, the mediator trust in physicians on the path from HIB to SDM can be altered by patients’ perceived healthcare quality. For instance, a patient who has a positive emotional response during HIB is likely to develop more trust in their physicians. This trust can be further enhanced if the patient also perceives the healthcare quality as high.

For utilization of social media in HIB, there is an interaction with perceived healthcare quality when the mediator is patients’ trust in sharing information with physicians (Figure 8). Our results indicate that high perceived healthcare quality enhances the positive effect of social media use on trust in sharing information, which, in turn, positively influences SDM frequency. However, we did not find a moderator role of patients’ perceived healthcare quality for the utilization of social media when the mediator is patients’ trust in physicians (Table 2). This indicates that, in the relationship between utilization of social media during HIB and SDM, patients’ trust in physicians is not changed by the levels of patients’ perceived healthcare quality. While the perception of healthcare quality can alter some of the relationships between HIB and SDM, mediated by trust, the critical role of trust and trust building between patients and physicians is further highlighted. Overall, this moderated mediation analysis not only offers robustness checking for our study, but also provides a more comprehensive understanding of the complex interplay between HIB, trust, healthcare quality perception, and SDM.

## 6. Limitations

We next highlight several limitations to this study. First, we relied on secondary data, which restricted our ability to customize measurements for key variables. For example, the data may not fully capture all aspects of HIB, trust, and SDM. Although we selected and combined all relevant items available, the survey was not originally designed to measure these constructs comprehensively. This may have limited our ability to fully capture the complexity of patients’ experiences. For future research, it is important to develop and utilize more precise and context-specific instruments to measure trust and other variables in healthcare settings. Moreover, our study uses simplified measures of trust, which is complex and multifaceted. Future work should explore more comprehensive, validated trust scales that account for its multidimensional nature. Lastly, we primarily focused on the frequency of SDM; however, equally important is the quality of SDM. The lack of quality-related measures in the dataset limits the scope of our conclusions. For a more comprehensive understanding of patient–physician collaboration, it is important to assess both the frequency and qualitative aspects of SDM, capturing both how often patients are involved and how well those decision-making experiences actually support them. Future research could address these limitations by employing more detailed measurements and methodologies to enrich our understanding of the relationship between HIB, trust, and SDM.

## 7. Conclusions

Exploring the relationship between patients’ health information behavior (HIB), shared decision-making (SDM), and trust in physicians is crucial for patient-centered healthcare. This study examines how trust bridges the gap between patients’ information behaviors and their engagement in decision-making processes. In the digital age, while patients have greater access to health information, challenges persist in ensuring information reliability and personalization. Trust is vital for guiding patients through information and enhancing SDM, ensuring decisions align with their preferences. Employing linear regression analysis, mediation analysis, and moderated mediation analysis, this research details the complex dynamics of trust, HIB, and SDM, enhancing patient empowerment and healthcare quality. It highlights the influence of emotional responses and social media use during HIB on trust and SDM, and the role of perceived healthcare quality in these relationships.

## Figures and Tables

**Table 1:** Description of items selected from factor analysis representing two categories.

Emotional Response During Health Information Behavior	Utilization of Social Media During Health Information Behavior
**I2.** Based on the results of your most recent search for information about cancer, how much do you agree or disagree: It took a lot of effort to get the information you needed (Strongly agree; Somewhat agree; Somewhat disagree; Strongly disagree)	**I10.** In the last 12 months, how often did you interact with people who have similar health or medical issues on social media or online forums? (Almost every day; At least once a week; A few times a month; Less than once a month; Never)
**(I3–5).** Based on the results of your most recent search for information about cancer, how much do you agree or disagree: **I3.** You felt frustrated during your search for the information (Strongly agree; Somewhat agree; Somewhat disagree; Strongly disagree)	**I11.** In the last 12 months, how often did you watch a health-related video on a social media site (for example, YouTube)? (Almost every day; At least once a week; A few times a month; Less than once a month; Never)
**I4.** You were concerned about the quality of the information (Strongly agree; Somewhat agree; Somewhat disagree; Strongly disagree)	**I12.** How much do you agree or disagree—I use information from social media to make decisions about my health (Strongly agree; Somewhat agree; Somewhat disagree; Strongly disagree)
**I5.** The information you found was hard to understand (Strongly agree; Somewhat agree; Somewhat disagree; Strongly disagree)	**I13.** How much do you agree or disagree—I use information from social media in discussions with my health care provider (Strongly agree; Somewhat agree; Somewhat disagree; Strongly disagree)

**Table 2:** Mediation analysis results for H2 and H3.

Mediator	HIB Dimension	Effect on SDM	Coefficient (β)	*p*-Value	Mediation Effect	Moderated Mediation
Trust in Physicians (H2.1)	Emotional Response	Direct Effect	0.683	<2 × 10^−16^	Partial Mediation (Proportion Mediated: 14.9%)	Significant (β = −0.079, *p* = 0.004)
		Indirect Effect	0.120	<2 × 10^−16^		
		Total Effect	0.803	<2 × 10^−16^		
	Utilization of Social Media	Direct Effect	−0.315	<2 × 10^−16^	Partial Mediation (Proportion Mediated: 9.53%)	Not significant (β = 0.061, *p* = 0.103)
		Indirect Effect	−0.033	0.0084		
		Total Effect	−0.348	<2 × 10^−16^		
Trust in Information Sharing (H2.2)	Emotional Response	Direct Effect	-	-	-	-
		Indirect Effect	-	-	-	-
		Total Effect	-	-	-	-
	Utilization of Social Media	Direct Effect	−0.409	<2 × 10^−16^	Partial Mediation (Proportion Mediated: −17.52%)	Significant (β = 0.087, *p* = 0.013)
		Indirect Effect	0.061	<2 × 10^−16^		
		Total Effect	−0.348	<2 × 10^−16^		

Note: The total effect is the effect of the HIB on SDM without considering the mediator. The direct effect and indirect effect represent the effects when controlling for the mediator. ‘Proportion Mediated’ indicates the percentage of the total effect that is mediated by trust. The moderator for the moderated mediation is perceived healthcare quality.

## Data Availability

Restrictions apply to the availability of these data. Data were obtained from the Health Information National Trends Survey (HINTS) and are publicly available at https://hints.cancer.gov/.

## Abbreviations

The following abbreviations are used in this manuscript:

SDM	Shared decision-making
HIB	Health information behavior
HINTS	Health Information National Trends Survey
EFA	Exploratory factor analysis

## Appendix A. Description of Items Initially Representing HIB

**I1.** Have you ever looked for information about cancer from any source? (Yes/No)	**I2.** Based on the results of your most recent search for information about cancer, how much do you agree or disagree: It took a lot of effort to get the information you needed (Strongly agree; Somewhat agree; Somewhat disagree; Strongly disagree)
**I3.** Based on the results of your most recent search for information about cancer, how much do you agree or disagree: You felt frustrated during your search for the information (Strongly agree; Somewhat agree; Somewhat disagree; Strongly disagree)	**I4.** Based on the results of your most recent search for information about cancer, how much do you agree or disagree: You were concerned about the quality of the information (Strongly agree; Somewhat agree; Somewhat disagree; Strongly disagree)
**I5.** Based on the results of your most recent search for information about cancer, how much do you agree or disagree: The information you found was hard to understand (Strongly agree; Somewhat agree; Somewhat disagree; Strongly disagree)	**I6.** In the past 12 months have you used the Internet to look for health or medical information? (Yes/No)
**I7.** How confident are you that you can find helpful health resources on the Internet? (Very confident; Somewhat confident; A little confident; Not confident at all)	**I8.** In the past 12 months, have you used a health or wellness app on your tablet or smartphone? (Yes/No)
**I9.** In the last 12 months, have you used an electronic wearable device to monitor or track health or activity? For example, a Fitbit, AppleWatch or Garmin Vivofit (Yes/No)	**I10.** In the last 12 months, how often did you interact with people who have similar health or medical issues on social media or online forums? (Almost every day; At least once a week; A few times a month; Less than once a month; Never)
**I11.** In the last 12 months, how often did you watch a health-related video on a social media site (for example, YouTube)? (Almost every day; At least once a week; A few times a month; Less than once a month; Never)	**I12.** How much do you agree or disagree—I use information from social media to make decisions about my health (Strongly agree; Somewhat agree; Somewhat disagree; Strongly disagree)
**I13.** How much do you agree or disagree—I use information from social media in discussions with my health care provider (Strongly agree; Somewhat agree; Somewhat disagree; Strongly disagree)	**I14.** In the past 12 months, did you receive care from a doctor or health professional using telehealth? (Yes/No)
**I15.** Have you ever used an app like Apple Health Records or CommonHealth to combine your medical information from different patient portals or online medical records into one place? (Yes/No)	**I16.** How many times did you access your online medical record or patient portal in the last 12 months? (0; 1 to 2 times; 3 to 5 times; 6 to 9 times; 10 or more times; do not have an online medical record or patient portal)
**I17.** In the past 12 months have you used your online medical record or patient portal to look up test results? (Yes/No)	**I18.** In the past 12 months have you used your online medical record or patient portal to view clinical notes (a health care providers written notes that describe your visit)? (Yes/No)

## Appendix B. Comparison of Regression Coefficients and Fit Metrics Across Imputation Methods

**Model**	**T1_1 Coef**	**T1_2 Coef**	**T2_1 Coef**	**T2_2 Coef**	**RSE**	**Adj R^2^**
Original Data	0.080	0.739	0.057	0.013	—	—
KNN Imputation	0.058	0.717	0.186	0.003	0.05	2.39
MICE Imputation	0.076	0.681	0.078	0.013	0.06	2.33
Random Forest	0.069	0.732	0.042	0.007	0.06	2.22
Note: To ensure robustness in handling missing data, we evaluated three imputation strategies: Multiple Imputation using Chained Equations (MICE), Random Forest Imputation (missForest), and k-Nearest Neighbors Imputation (KNN). The coefficients across all four models show consistent directions and significance patterns. The KNN-imputed model shows the lowest Residual Standard Error (RSE = 0.05) and the highest Adjusted R^2^ (2.39), suggesting that it explains more variance with lower residual noise than other methods. Importantly, the core relationships between trust indicators and SDM remain stable across models, indicating that the imputation did not alter the key findings of the analysis. This supports the robustness of the results and validates the use of KNN for handling missing data.						

## Appendix C. Controlled Demographic Information

**Variable**		**n**	**Percentage**
Birth gender	Male	2353	37.64%
	Female	3899	62.36%
Age	18–34	966	15.45%
	35–49	1279	20.46%
	50–64	1799	28.77%
	65–74	1361	21.77%
	75+	847	13.55%
Education	Less than high school	448	7.17%
	High school graduate	1210	19.35%
	Some college	1672	26.74%
	Bachelor	1808	28.92%
	Post-baccalaureate	1114	17.82%
Race	Hispanic	1194	19.10%
	Non-Hispanic White	3203	51.23%
	Non-Hispanic Black	1032	16.51%
	Non-Hispanic Asian	626	10.01%
	Non-Hispanic other	197	3.15%
Ever Had Cancer	Yes	1084	17.34%
	No	5168	82.66%

## Appendix D. Detailed Results of Factor Analysis

*Step 1: Determine Number of Factors to Extract*

Using Kaiser’s rule, the number of factors is equal to the number of factors with eigenvalues greater than 1.0. We obtained five factors first.

*Step 2: Factor analysis with fa() function from package GPArotation*

This is typically employed for conducting exploratory factor analysis (EFA). Since we did not have a predefined notion of the structure or number of factors in information seeking and management experiences from the HINTS data, we started with all the suitable survey questions and determined the number of factors that best explain the correlation among the variables.

Based on the above results, we removed I9, I14, and I15. Additionally, we needed to carefully review the content of I1, I6, I8, I10, I11, I12, and I13 since multiple cross-loading exists. We determined if these items conceptually make sense when grouped together due to the complex factor structure with overlapping items.

*Step 3: Refine the Factor Model with Iterative Process*

We removed factors that did not load well in the first round after reducing it to three categories (I17, I18), trying to reach a clearer, more interpretable factor structure. Additionally, we wanted to have higher loadings for each variable (like above 0.5 or 0.6) to be stronger and more desirable. Then, we removed I16 first, which had the lowest loading of 0.3, repeating the steps and reducing the categories.

The emotional response during HIB factor was constructed from four items (I2, I3, I4, I5), reflecting patients’ emotional reactions during HIB. Each item was standardized, with higher scores indicating more positive emotional responses and lower scores suggesting negative ones (e.g., strong agreement with statements of effort, frustration, concern, or difficulty in understanding). The overall internal consistency was 0.87, as indicated by the Cronbach’s alpha score (eigenvalue = 2.88; factor loading = 0.7–0.9).

The utilization of social media during HIB factor was represented by four items (I10, I11, I12, I13) that assessed the degree of patients’ engagement with social media for health-related purposes. That is, higher scores represented more utilization of such platforms, whereas lower scores indicated less engagement. The overall internal consistency was 0.69, as indicated by the Cronbach’s alpha score (eigenvalue = 2.07; factor loading = 0.5–0.7).
